# Shared Control Schemes for Middle Ear Surgery

**DOI:** 10.3389/frobt.2022.824716

**Published:** 2022-03-22

**Authors:** Jae-Hun So, Stéphane Sobucki, Jérôme Szewczyk, Naresh Marturi, Brahim Tamadazte

**Affiliations:** ^1^ CNRS UMR 7222, INSERM U1150, ISIR, F-75005, Sorbonne Université, Paris, France; ^2^ Extreme Robotics Lab, University of Birmingham, Birmingham, United Kingdom

**Keywords:** medical robotics, tele-operation, comanipulation, visual servoing, robot control

## Abstract

This paper deals with the control of a redundant cobot arm to accomplish peg-in-hole insertion tasks in the context of middle ear surgery. It mainly focuses on the development of two shared control laws that combine local measurements provided by position or force sensors with the globally observed visual information. We first investigate the two classical and well-established control modes, i.e., a position-based end-frame tele-operation controller and a comanipulation controller. Based on these two control architectures, we then propose a combination of visual feedback and position/force-based inputs in the same control scheme. In contrast to the conventional control designs where all degrees of freedom (DoF) are equally controlled, the proposed shared controllers allow teleoperation of linear/translational DoFs while the rotational ones are simultaneously handled by a vision-based controller. Such controllers reduce the task complexity, e.g., a complex peg-in-hole task is simplified for the operator to basic translations in the space where tool orientations are automatically controlled. Various experiments are conducted, using a 7-DoF robot arm equipped with a force/torque sensor and a camera, validating the proposed controllers in the context of simulating a minimally invasive surgical procedure. The obtained results in terms of accuracy, ergonomics and rapidity are discussed in this paper.

## 1 Introduction

The tasks performed by robots are becoming more and more complex and are no longer limited to positioning or pick and place. A significant amount of progress has been made to increase the autonomy of robots, especially in certain industries like automotive, aerospace etc. However, several scientific and technological obstacles persist to reach a sufficient degree of autonomy to carry out more complex tasks, particularly in unknown, unstructured and dynamic environments. Modern-day robots are expected to share the workspace with humans or other robots in a safe and secure manner (Burgard et al., 2000; Roy and Dudek, 2001). To this extent, external sensors such as force torque sensors, cameras, lidars etc. play a prominent role.

For instance, force sensors provide local contact information while handling or grasping objects during insertion and assembling tasks; vision sensors such as cameras provide rich and global information (which can also be local in some cases) of the robot environment (Espiau et al., 1992; Chaumette et al., 2016). This brings up the question, whether it is possible to effectively combine several modes of perception (e.g., force and vision) within the same control scheme to benefit from their complementary advantages? Of course, this does not concern the sequential controllers that are usually reported in the literature (Baeten et al., 2003). In general, the robot is first controlled using the visual information provided by the camera(s) when the end-effector is far from the target, and then switches to the proximity sensing (e.g., position or force), when the robot is close or in contact with the target. In most cases, it is trivial to combine measurements from different sensors such as position and force in the same control scheme (Morel et al., 1998; Mezouar et al., 2007; Prats et al., 2007; Liang et al., 2017; Adjigble et al., 2019). However, fusion of information, e.g., provided by the camera and the force sensor, is highly challenging due to the fact that these features are fundamentally different. Very few works have studied this possibility (Morel et al., 1998; Mezouar et al., 2007) and more recent works showed interest on this topic (Abi-Farraj et al., 2016; Selvaggio et al., 2018; Adjigble et al., 2019; Selvaggio et al., 2019).

This paper focuses on the development of two shared control laws, which combine proximal (local) and global measurements. Specifically, local measurements provided by position or force sensors are combined with globally observed visual information, which is acquired from a camera attached to the robot end-effector in an *eye-in-hand* setting. In our previous work, (So et al., 2020; So et al., 2022), have demonstrated how a robotic arm can be controlled in a fully and well-established position-based manner by means of tele-operation with a Phantom Omni system. There, we have developed an end-frame tele-operation controller. This approach has shown promising performances in terms of accuracy, repeatability and intuitiveness, particularly in the context of minimally invasive surgery. However, we have observed a strong coherence between the overall performance and operator’s expertise levels.

Thus, in many cases, in particular if the operator is a surgeon, it turned out to be highly challenging to control all the degrees of freedom (DoF) of the surgical instrument (e.g., attached to robot’s end-effector) merely using a joystick. Apparently, handling end-effector spatial rotations was particularly difficult for a surgeon who has to focus at the same time on the surgical procedure. Similar conclusions were drawn for the other control scheme presented in (So et al., 2020), which uses comanipulation mode of the robotic arm.

Addressing the aforementioned issues with our previous work, in this paper, we propose a new shared controller where a robot arm can be controlled either fully force-based or by using a shared control law that combines force and vision measurements in the same control loop. The operator, *i.e.*, a surgeon controls the linear (translational) axes of the tool via a human-robot interface while the rotations are automatically controlled by the vision-based controller. Thus, we first expressed two well-established controllers. The first one consists of a position-based end-frame tele-operation controller and the second one is a comanipulation controller. The main contributions of this paper lie in the formulation of new hybrid controllers by consistently combining two physical quantities in the control loop. This allows separating the linear DoF which are controlled in tele-operation (respectively, comanipulation) with the rotational ones that are controlled simultaneously and intuitively by a vision-based controller. To this extent, four shared control modes are studied and evaluated, which are listed as follows: 1) parallel hybrid tele-operation; 2) external hybrid tele-operation; 3) parallel hybrid comanipulation; and, external hybrid comanipulation. These are presented in detail in Section 3.

The developed controllers are experimentally validated using a 7-DoF robot arm, in the context of simulating a minimally invasive surgical procedure. We have conducted subjective analysis by recruiting two different groups of people to study and compare the performance of various controllers in terms of accuracy, behaviour, intuitiveness, and the time required to accomplish a target task. First group of people are the novice operators, who have never used the system; and, the second group consists of expert operators, who have developed the system and possess prior experience operating it. In this work, we compare the performance of conventional teleoperation and comanipulation controllers presented in (So et al., 2020) with our proposed methods. It has been demonstrated that the proposed shared controllers outperform the classical ones in multiple points, e.g., ergonomy, accuracy, etc.

The rest of this paper is organized as follows. In Section 2, we discuss the clinical framework of this work. Section 3 deals with the methodology followed to carry out the proposed controllers as well as with the motivations that led us to develop these controllers. Section 4 presents the implemented experimental setup to assess the different controllers and compare them to the classical tele-operation and comanipulation control modes. A discussion on the pros and cons of each control approach is also reported in this section.

## 2 Clinical Motivations and Middle Ear Surgery Requirements

### 2.1 Motivation

The work discussed in this paper is part of a larger research and development investigation focusing on an ergonomic and intuitive robotic solutions for middle ear surgery (Dahroug et al., 2017; Dahroug et al., 2018; Dahroug et al., 2020; So et al., 2020). The primary challenge concerns the design and fabrication of a macro-and microscale robotic system, which consists of a conventional collaborative robotic arm fixed with a miniature continuum robot as the end-effector. The tool can enter and operate inside the middle ear through a millimetric-sized incision made in the mastoid. The secondary challenge is to develop intuitive and ergonomic control modes allowing the surgeon to perform several tasks during surgery such as bringing an instrument close to the surgical site, insert it into the middle ear cavity through an entry incision hole, position the surgical tool in front of pathological tissues for mechanical resection or laser burring process (Hamilton, 2005).

**FIGURE 1:**
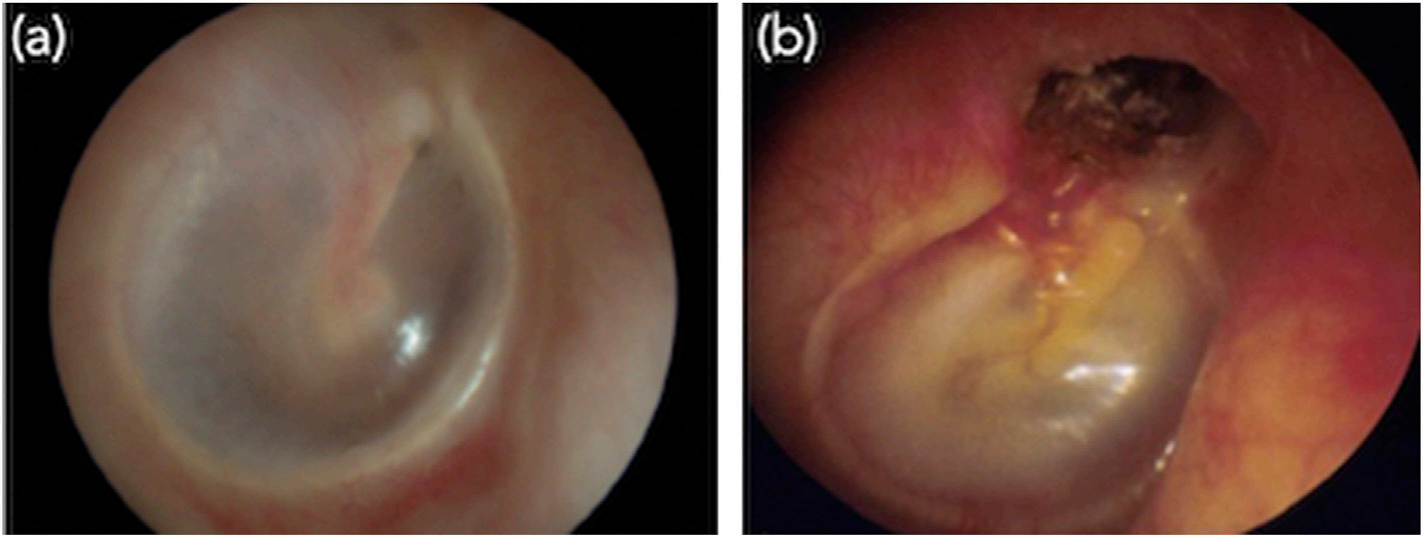
Images of the internal of middle ear. **(A)** Normal ear and **(B)** typical primary acquired cholesteatoma, which destroyed the tympanic membrane.

The typical surgical treatment we consider in this work involves the total removal of a pathological tissue that develops inside the middle ear cavity known as cholesteatoma (Holt, 1992). Cholesteatoma can be defined as an abnormal skin growth that occurs in the middle ear cavity, see Figure 1. It is usually due to blocked ventilation where dead skin cells cannot be ejected out of the ear. Gradually, the cholesteatoma expands in the middle ear, filling in the empty cavity around the ossicles and then eroding the bones themselves (ossicles and mastoid). Cholesteatoma is often infected and results in chronically draining ears. It also results in hearing loss and may even spread through the skull-base into the brain. It was reported that one in 10,000 citizens are diagnosed with cholesteatoma every year in the world (Olszewska et al., 2004; Møller et al., 2020).

Currently, the most effective treatment for cholesteatoma is to surgically resect or ablate the infected tissues (Derlacki and Clemis, 1965; Bordure et al., 2005). The current surgical procedure raises at least two major issues. The first is the degree of invasiveness required for such procedure. To get access to the middle ear cavity, the surgeon performs an incision hole of about 20 mm diameter in the mastoid. Through this, the surgeon visualizes the cholesteatoma as well as pass tools to treat the pathological tissues. The second limitation concerns the clinical efficiency of this procedure, which never ensures an exhaustive removal of the pathological tissues.

Moreover, there is not much literature on aggregate data (by region or country) on the recurrence of cholesteatoma with current surgical methods. However, it has been reported in studies conducted by health care institutions that depending on the surgeon expertise, approximately 25% of cholesteatoma treatments are unsuccessful leading to cholesteatoma persistence or recurrence (Aquino et al., 2011; Møller et al., 2020). These limitations of the current surgical procedures can be tackled by the design of the new micro-and macroscale robotic solution and the development of dedicated control architectures that will make the procedure less invasive and more efficient.

### 2.2 System Requirements

In one of our previous works (Dahroug et al., 2018), we have evaluated the robotic systems that are currently used for middle ear surgery as well as the control modes that are implemented on these systems. Due to various limitations associated with the existing approaches, we have envisioned the characteristics of an ideal system and the control modes that will improve current practices in cholesteatoma surgery. As observed in (Dahroug et al., 2018), we will need a robotic arm with a minimum of 4-DoFs, a workspace of 1,000 mm × 1,000 mm × 500 mm, a spatial accuracy of at least 0.05 mm. It has been also perceived that the robotic system must be equipped with exteroceptive vision and force sensors. Besides, the control modes considered for surgical protocol are tele-operation, comanipulation, semi-automatic with operator intervention, and full-automatic mode for certain surgical sub-tasks (e.g., laser ablation of the residual cholesteatoma cells).

## 3 Shared Controllers

**FIGURE 2:**
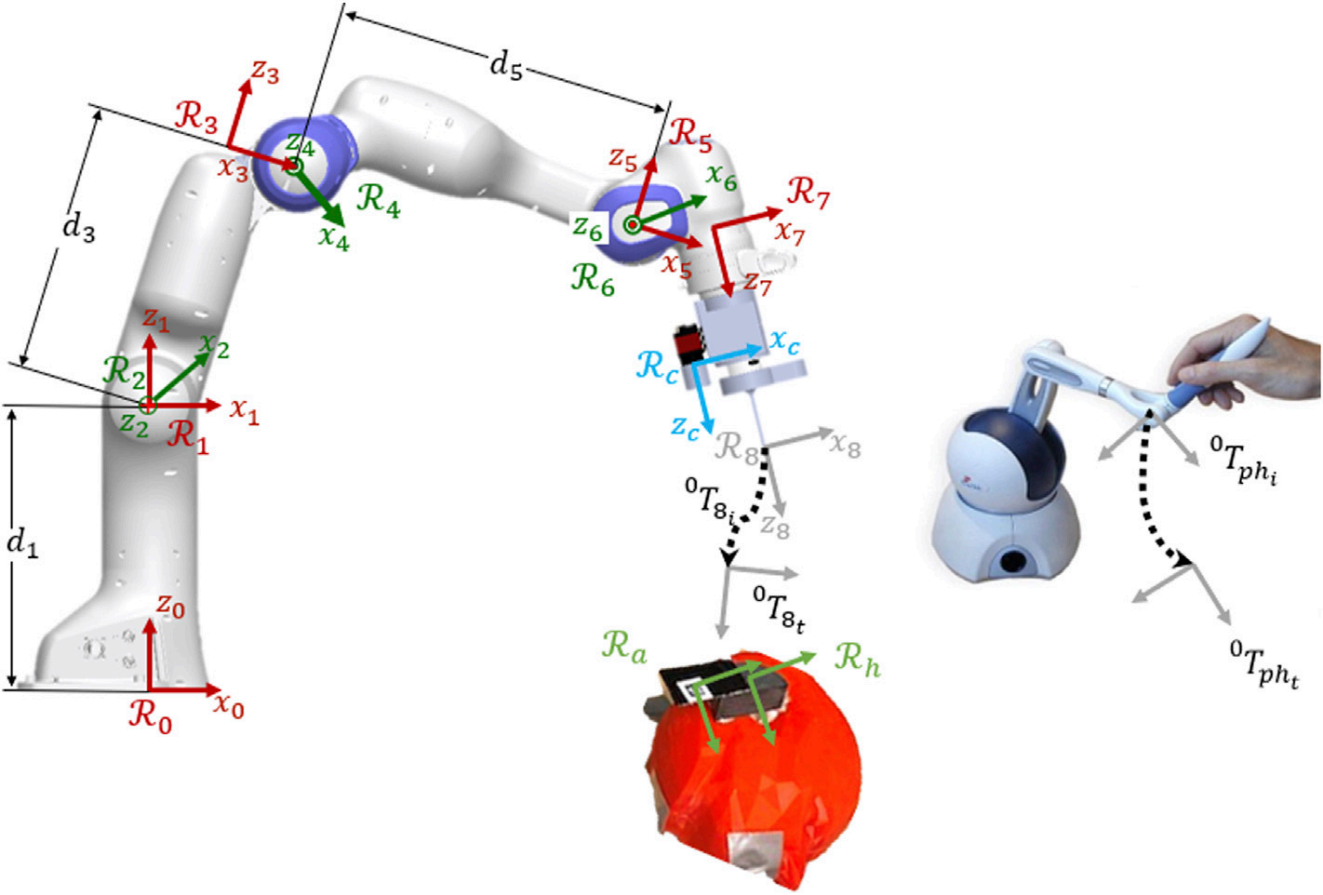
Kinematic model of the robotic system with the associated frames, the joystick for the teleoperation and the target positions (marker and incision hole).

Before discussing the proposed control modes, in this section, we first present the technical details of the classical tele-operation and comanipulation approaches. Following, the proposed hybrid tele-operation and comanipulation methods are detailed. Figure 2 depicts the robotic system, the robot arm and the joystick with their associated frames and the notations that are used in the following. The incision hole and the fiducial marker are also present in the figure with the associated frames, representing the target position of the tool-tip and the camera FoV.

### 3.1 Classical Tele-Operation

Classical tele-operation is widely used for a variety of robotic applications (Simorov et al., 2012). The tele-operation mode developed in this work is based on a position-based controller allowing the interpretation of the local pose of a Phantom Omni end-effector as the desired pose of the tool tip attached to the robot end-effector. The motion of the joystick is directly mapped into the robotic system’s end-effector space, which gives an intuitive position-based control for the user.

**FIGURE 3:**
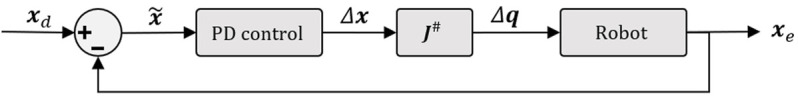
Control scheme of the classical tele-operation.

To control the robot by tele-operation, the desired position ***x***_*d*_ of the joystick expressed in frame $R_{8}$ is used as the control input (Figure 3). Using the continuous mapping between robot end-effector motion and that of the joystick, the position error $\tilde{x}$ (on the end-effector’s position) is expressed as

$$\tilde{x}=x_{d}-x_{e}$$ (1)

where, ***x***_*e*_ is robot end-effector positions. $\tilde{x}$ goes through a proportional-derivative (PD) controller in order to get the control set-point Δ ***x***. Therefore, the robot joint space velocities Δ ***q*** are obtained from the task-space velocities using the robot’s Jacobian pseudo-inverse $J^{\#}\in R^{8\times 6}$ :

$$\Delta q=J^{\#}\Delta x$$ (2)

Note that internal movements of the robot arm are not exploited in our approach and Δ ***q*** obtained from (2) is simply the minimum norm solution of the robot inverse differential kinematics.

### 3.2 Classical Comanipulation

Another classical strategy to control the motion of aforementioned robotic system is comanipulation. Such a technique has been widely used in the surgical domain to accomplish a variety of tasks (Zhan et al., 2015). In this work, we have implemented a comanipulation scheme on our robotic system using an external 6-DoF Force/Torque (F/T) sensor. In the presented system, the force sensor is attached to the robotic arm at the frame $R_{8}$ where the control point is located. The implemented comanipulation mode is trivial and does not require any registration or specific parameters tuning.

**FIGURE 4:**
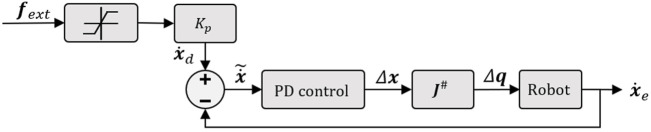
Illustration of the implemented control scheme in case of comanipulation mode.

To move the robot in the comanipulation manner, an ergonomic 3D printed wrist is provided to the user (see experimental setup Figure 7 for details). The wrist is attached to the F/T sensor, which is attached to the distal part of the robotic arm. The user pushes the wrist and the F/T sensor provides the applied force and torque onto the wrist, represented by ***f***_*ext*_, expressed in the frame $R_{8}$ . The measured force and torque are then converted to desired operational velocities, represented by ${\dot{x}}_{d}$ , thanks to a proportional gain and min/max filter and the output is injected into a PD controller (Figure 4). Finally, (2) allows converting the task-operational control vector Δ ***x***_8_ to joint-space operational one Δ ***q***_8_.

### 3.3 Developed Shared Controllers

The two control modes discussed above are functional and relatively suitable for performing a large number of tasks in an operating room. However, in order to make the controllers more intuitive and easier to use, especially for a non-specialist (e.g., a novice surgeon), we have developed shared controllers. Indeed, it has been shown that among the 6 DoFs involved in a 3D positioning task, rotations (i.e., *θ*_*x*_, *θ*_*y*_, *θ*_*z*_) are the most difficult to achieve, in particular for a non-specialist (Adjigble et al., 2019). Accordingly, to reduce the cognitive load on the surgeon, we have proposed two decoupled control modes allowing to dissociate translations and rotations. Thereby, the three involved rotations will be managed automatically based on the vision feedback, when the three translations are still controlled by the introduced classical tele-operation or comanipulation modes.

#### 3.3.1 Parallel Hybrid

**FIGURE 5:**
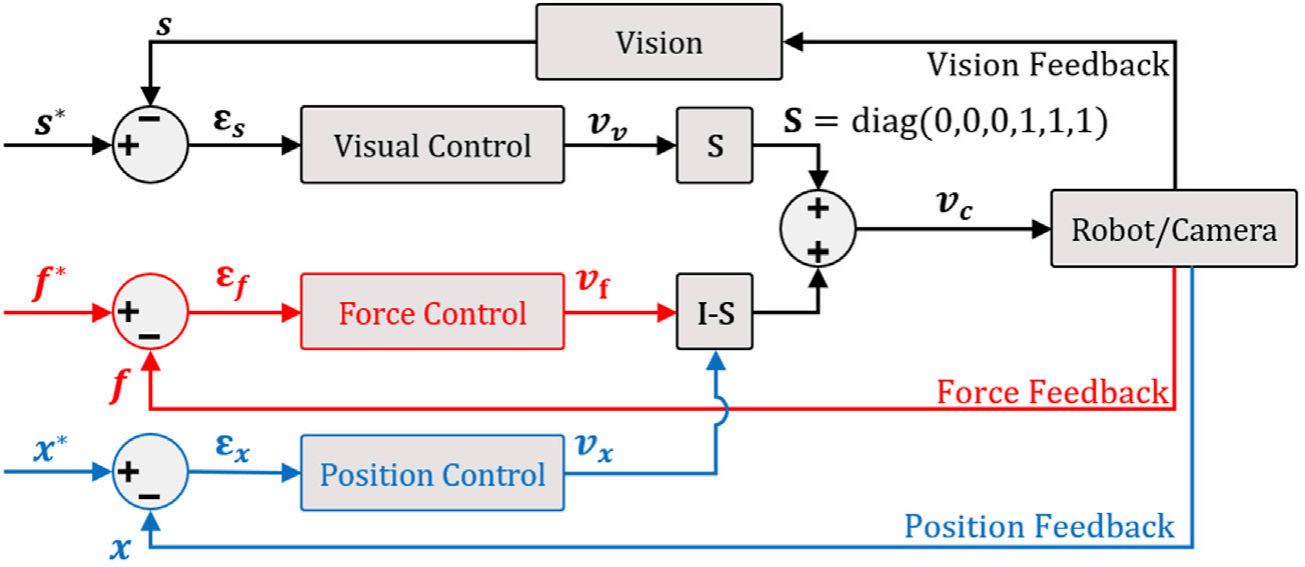
Parallel hybrid force/vision comanipulation (in red) and position/vision teleoperation (in blue) control scheme.

The first shared controller we develop in this work is the parallel hybrid. It consists of the parallel juxtaposition of two internal loops. The first is a vision feedback loop to manage automatically the angular motion of the robot. The second is a position-based (in case of tele-operation mode) or force-based (in case of comanipulation) loop to control the linear motion. Figure 5 shows the architecture of the so-called hybrid shared controllers.

To have a decoupled control law, i.e., rotations and the translation are controlled independently without one interfering with the other and then avoiding any conflict at the actuator level, we have opted for a position-based visual controller (Chaumette and Hutchinson, 2006). Thereby, to ensure the orthogonality between force or position and vision controller outputs, it is essential to introduce a selection matrix S=diag(0,0,0,1,1,1) . The selection matrix allows selecting the rotation DoFs to be managed by the vision-based control and the remaining DoFs, i.e., translations to be controlled by the force or position controller.

As previously stated, the context of this work is to execute a middle ear surgery wherein the target corresponds to an incision of millimeter-diameter performed in the mastoid (access to the middle ear cavity). Following the usual way for registration and surgery, a printed *AprilTag code* with known geometry is fixed on the mastoid (Katanacho et al., 2016). Using this, we estimate the full pose of the target (orientations and translations) in the camera frame $R_{c}$ . It is worth noting that the homogeneous transformation from the frame $R_{h}$ attached to the incision hole towards the camera frame $R_{c}$ is represented by Mic .

A feature characterizing the camera-hole relative configuration can be extracted from Mic as ***s*** = (***t***, *θ* ***u***)^*⊤*^, where ***t*** is the translational part ${\left(t_{x},t_{y},t_{z}\right)}^{⊤}$ of the homogeneous matrix Mic and *θ* ***u*** is the axis-angle representation of the rotational part. Furthermore, to regulate the Cartesian error ***e*** between the current position of the robot end-effector noted ***r*** and the desired position ***r*** *, corresponding to configuration when the tool is inserted in the incision hole. By considering ***s*** = (***t***, *θ* ***u***)^*⊤*^ as the current position and $s^{*}={\left(t^{*},\;0_{1\times 3}\right)}^{⊤}$ as the desired one, then the Cartesian error ***e*** is

$$e=s^{*}-s={\left(\Delta t,-\theta u\right)}^{⊤}$$ (3)

where, Δ ***t*** = ***t*** * − ***t***. The time-derivation of ***s***, i.e., $\dot{s}={\left(\dot{t},\;\dot{\theta }u\right)}^{⊤}$ allows linking the camera velocity twist ***v*** = (*v*,*ω*)^*⊤*^ to the visual features variation (Marchand et al., 2002):

$$\dot{s}=\left(\begin{array}{cc} I_{3\times 3} & 0_{3\times 3}\\ 0_{3\times 3} & L_{w} \end{array}\right)\left(\begin{array}{c} v\\ \omega \end{array}\right)$$ (4)

where,

$$L_{w}=I_{3\times 3}-\frac{\theta }{2}{\left[u\right]}_{\times }+\left(1-\frac{\text{sinc}\theta }{{\text{sinc}}^{2}\frac{\theta }{2}}\right){\left[u\right]}_{\times }^{2}$$ (5)

in which ***I***_3×3_ is the 3 × 3 identity matrix and ***L***_*w*_ is the interaction matrix such that $L_{w}^{-1}\theta u$ = *θ* ***u*** as reported in (Malis et al., 1999). Note that the notation *θ* ***u*** represents the angle-axis parameterization of the rotation, $\text{sinc}\left(\theta \right)=\frac{sin\left(\theta \right)}{\theta }$ , and [***u***]_×_ being the anti-symmetric matrix associated to the vector ***u***. Therefore, the camera velocity twist can be expressed as follows:

vω=−λt−t*θu (6)

Finally, the selection matrix S allows expressing the parallel hybrid controller as follows: *v* = *v*_*f*_ (provided by the force controller) and *ω* = − *λθ* ***u*** (provided by the vision controller).

#### 3.3.2 External Hybrid

**FIGURE 6:**
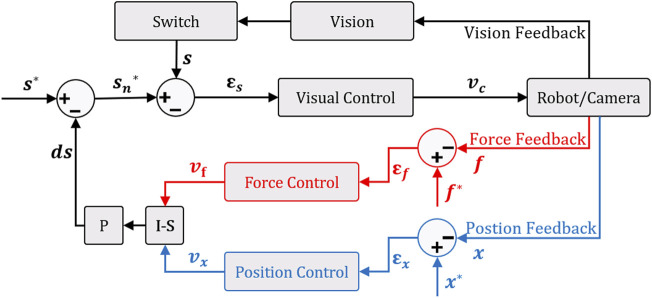
External hybrid force/vision comanipulation (in red) and position/vision tele-operation (in blue) control scheme with the function of keeping the target in the center of the camera field-of-view.

When both the force/position and vision-based controllers work in parallel, there is a risk that the tracked visual features (i.e., the *AprilTag*) are lost. This could jeopardize the accuracy of final positioning task. To tackle this issue, we have designed a new hybrid controller as shown in Figure 6. The underlying idea is to express the control task as two hierarchical sub-tasks. The first task (priority sub-task) deals with maintaining the target (i.e., *AprilTag*) at the center of the camera field-of-view (FoV), while the second one (secondary sub-task) is devoted to the regulation of the error between the current and the desired poses. 

Therefore, to continuously maintain the *AprilTag* in the center of the camera FoV, we introduce a target locking mode based on the angular deviation *θ* ***u***_*lock*_ that can be defined as follows:

$$\begin{array}{ccc} \theta u_{lock_{x}} & = & \text{atan}2\left(t_{y},t_{z}\right)\\ \theta u_{lock_{y}} & = & \text{atan}2\left(t_{x},t_{z}\right) \end{array}$$ (7)

where, *t*_*x*_, *t*_*y*_, and *t*_*z*_ are respectively the translation components along the *x*, *y*, *z* directions of the homogeneous matrix Moc that represents the 3D pose of the *AprilTag* fiducial marker expressed in the camera frame $R_{c}$ . In the target locking mode, the rotation part of the desired position is then defined as $\theta u_{lock}=\left(\theta u_{lock_{x}},\theta u_{lock_{y}},\theta u_{z}\right)$ . Besides, the Euclidean distance $e_{xy}=\sqrt{t_{x}^{2}+t_{y}^{2}}$ expressed in the camera frame $R_{c}$ , is used to define a threshold to switch between free and locked modes:

$$s\left(e_{xy}\right)=\left\{\begin{array}{cc} s={\left(0_{1\times 3},\;\theta u_{lock}\right)}^{⊤},\; & \text{if }e_{xy}>\epsilon =\sqrt{\frac{1}{2}}d\\ s={\left(0_{1\times 3},\;\theta u\right)}^{⊤},\; & \text{else} \end{array}\right.$$ (8)

where, *d* is the side-size of the *AprilTag* and *ϵ* is a predefined threshold triggering the switch between free and locked modes.

In this external hybrid force (or position) vision controller, the controller outputs are used to modify the desired visual features vector ***s*** *. This is associated in the visual servo control by considering that the desired visual features is $s_{n}^{*}$ = ***s*** * - ***ds*** = ${\left(t^{*}-t_{ds},\;0_{1\times 3}\right)}^{⊤}$ and the Cartesian error is $e=s_{n}^{*}-s\left(e_{xy}\right)$ . Thereby, the final camera velocity twist is expressed as follows:

vω=−λtds−t*θuexy (9)

then,

$$\begin{array}{ccc} v & = & -\lambda \left(t_{ds}-t^{*}\right) \end{array}$$

$$\begin{array}{ccc} \omega & = & \left\{\begin{array}{cc} -\lambda \theta u_{lock},\; & \text{if }e_{xy}>\sqrt{\frac{1}{2}}d\\ -\lambda \theta u,\; & \text{else} \end{array}\right. \end{array}$$ (10)

Our system is designed in an *eye-in-hand* configuration. Thus, the relation between the robot velocity $\dot{q}$ and the camera velocity ***v*** is obtained as follows:

q˙=−J#Vc0v (11)

where ***J*** ^***#***^ is the pseudo-inverse kinematic Jacobian matrix of the 7-DoF robot arm in the base frame $R_{0}$ , and Vc0 is the transformation matrix associated to the velocity change frame from $R_{c}$ to $R_{B}$ constructed as:

Vc0=Rc0tc×0Rc00Rc0

where Rc0 is the 3 × 3 rotation matrix from $R_{c}$ to $R_{B}$ , tc0 is the 3 × 1 associated translation vector, and tc×0 is the skew symmetric matrix associated to the vector cross-product.

## 4 Experimental Validation

### 4.1 Experimental Setup

**FIGURE 7:**
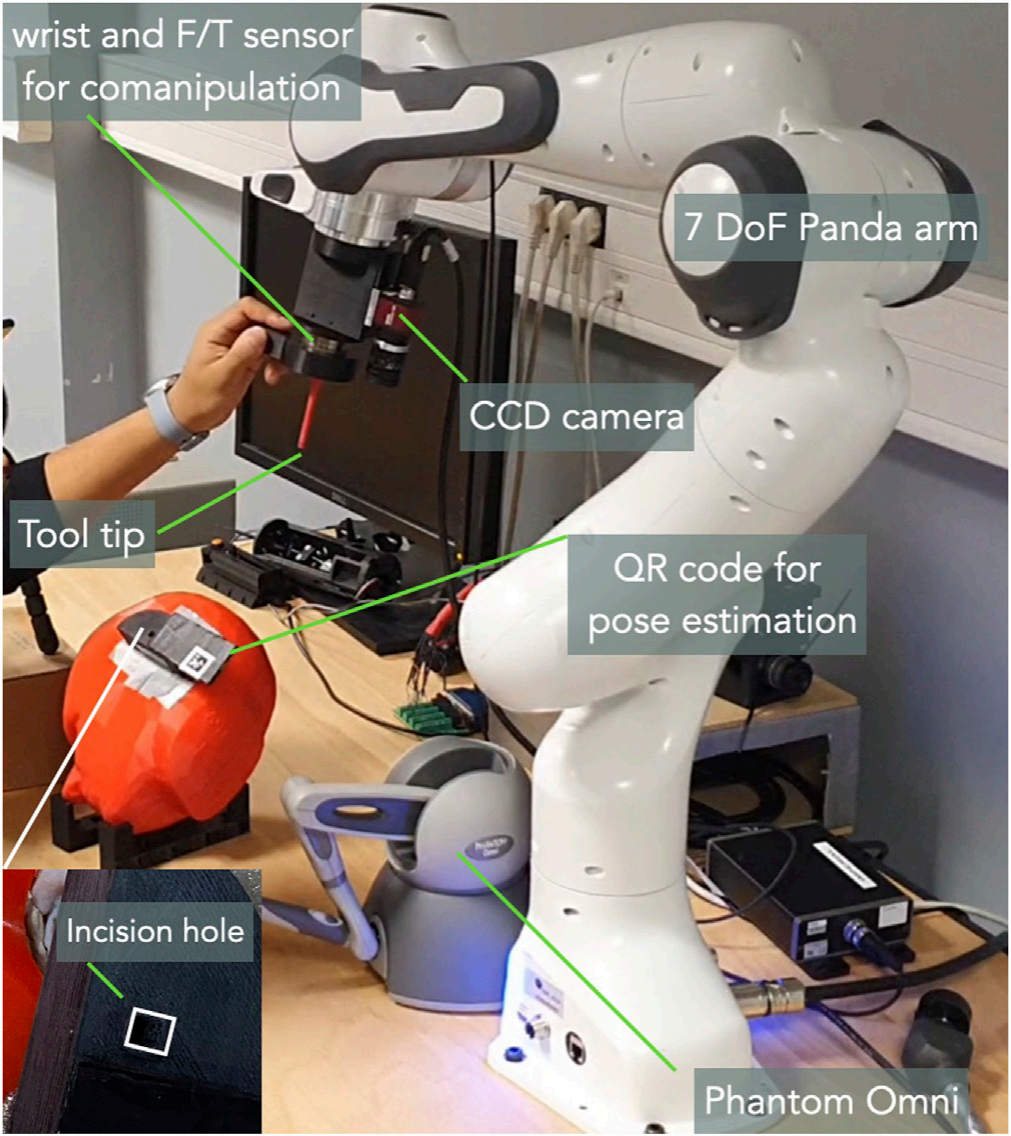
The developed experimental setup for this work.

To assess the proposed controllers, we have developed an experimental setup consisting of a 7-DoF cobot (FRANKA EMIKA Panda), as shown in Figure 7. A 3D printed tool (2 mm of diameter), which mimics a typical surgical instrument used to operate middle ear, is fixed as an end-effector of the robot. A standard CCD camera, AVT Guppy PRO F033b (resolution: 800 × 600 pixels and frame-rate: 25 images/second), is mounted in an *eye-in-hand* configuration on the robot end-effector. A Sensable Phantom Omni haptic device is used to teleoperate the robotic system. For the sake of comanipulation, a 6-DoF force/torque sensor (ATI MINI-40) is fixed at the robot distal part. A head phantom (Figure 7) at scale 1 : 1 is positioned to simulate the position of a patient on the operating table. Finally, the tunnel drilled on the head has the shape of the 3D tool fixed on the robot with a tolerance of 0.1 mm. It is worth noting that an *AprilTag QR-code* marker is positioned next to the incision hole, with a distance equal to that of between the tool-tip and the camera. This is to maintain the *AprilTag* inside the camera’s FoV until the end of insertion task.

### 4.2 Validation Scenario

The work is validated by analysing the scenario where a 3D printed tool is inserted into an incision hole. Initially, the robot is placed in an arbitrary position, then the operator jogs it towards the incision so as to insert the tool. The depth of the incision hole is estimated to be 5 *mm*. A small line was drawn at 5 *mm* before the tool-tip. A group of 5 participants (2 expert and 3 novice) are volunteered to carry out the positioning and insertion tasks using different tele-operation (classic, parallel hybrid and external hybrid) and comanipulation (classic, parallel hybrid and external hybrid) control modes. For each of the performed tasks, Cartesian errors (along each DoF), the twist ***v***_*c*_ expressed in the camera frame, the 3D trajectory of the robot end-effector, as well as the time required to achieve the tasks are recorded and analysed.

### 4.3 Experimental Results

As stated above, the insertion task was performed by 5 different participants. Every participant was asked to repeat the same task three times, alternatively using the developed controllers (3 control modes for the tele-operation and 3 others for the comanipulation).

#### 4.3.1 Accuracy

The first analysis metric concerns the accuracy of each control mode. The final error *e*_*f*_ corresponds to the difference between the final position of the robot end-effector and the reference position (recorded when the 3D printed tool is inserted into the incision hole).

**FIGURE 8:**
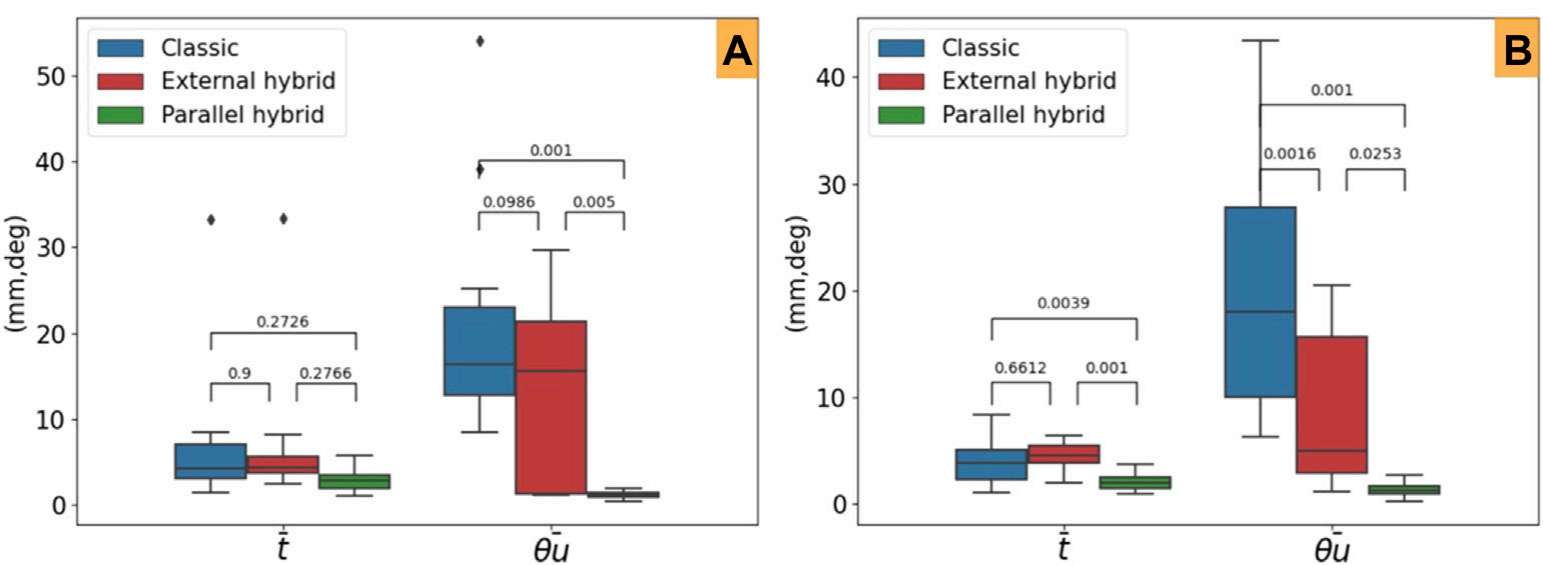
Mean steady-error and post-hoc Tukey HSD *p*-values for the evaluated control laws. **(A)** Tele-operation modes and **(B)** comanipulation ones.

Table 1 summarizes the Cartesian error for each DoF. It can be noticed that the classical tele-operation mode is slightly more accurate than the external hybrid one. However, the parallel hybrid mode is very accurate (Figure 8A) with a mean linear error (average of *e*_*x*_, *e*_*y*_, and *e*_*z*_) estimated to be 0.94 mm (2.24 and 4.17 mm for the classical and external hybrid methods, respectively), while the mean angular error (average of $e_{\theta_{x}}$ , $e_{\theta_{y}}$ , and $e_{\theta_{z}}$ ) is 0.17° (6.74° and 4.27° for the classical and external hybrid methods, respectively).

**TABLE 1:** Comparison of the positioning errors obtained with different control modes.

Control	*e*_*x*_ (mm)	*e*_*y*_ (mm)	*e*_*z*_ (mm)	$e_{\theta_{x}}$ (deg)	$e_{\theta_{y}}$ (deg)	$e_{\theta_{z}}$ (deg)
CTo1	1.79 ± 1.57	1.21 ± 1.09	3.47 ± 6.84	6.88 ± 3.56	4.18 ± 3.45	9.16 ± 10.07
**PHTo**2	***0.78*** ***±*** ***0.55***	***1.22*** ***±*** ***0.93***	***0.83*** ***±*** ***0.77***	***0.34*** ***±*** ***0.17***	***0.31*** ***±*** ***0.24***	***0.5*** ***±*** ***0.28***
EHTo3	2.51 ± 3.65	1.39 ± 1.11	2.61 ± 4.79	6.46 ± 6.83	5.6 ± 5.93	0.76 ± 0.62
CCo4	0.73 ± 0.6	1.73 ± 1.09	1.52 ± 1.18	3.67 ± 3.33	7.49 ± 4.14	8.87 ± 9.22
**PHCo**5	***0.49*** ***±*** ***0.52***	***0.97*** ***±*** ***0.5***	***0.55*** ***±*** ***0.52***	***0.37*** ***±*** ***0.27***	***0.46*** ***±*** ***0.34***	***0.52*** ***±*** ***0.31***
EHCo6	1.16 ± 0.47	2.19 ± 0.96	1.12 ± 0.69	1.84 ± 1.62	5.88 ± 5.7	1.49 ± 1.37

Bold represents the best performance obtained in terms of accuracy on all degrees of freedom (translations and rotations) when comparing the different proposed control modes. *Tukey HSD *p*-value (tele-operation, mean angular error): classical *vs* external hybrid → *p* = 0.005 and classical *vs* parallel hybrid → *p* = 0.001. Note that *p* < 0.05, which means statistically significant.

*Tukey HSD *p*-value (comanipulation, mean angular error): classical *vs* external hybrid → *p* = 0.001 6 and classical *vs* parallel hybrid → *p* = 0.001.

* Tukey HSD *p*-value (comanipulation, mean linear error): classical *vs* external hybrid → *p* = 0.001 and classical *vs* parallel hybrid → *p* = 0.003 9.

a CTo, classical tele-operation.

b PHTo, parallel hybrid tele-operation.

c EHTo, external hybrid tele-operation.

d CCo, classical comanipulation.

e PHCo, parallel hybrid comanipulation.

f EHCo, external hybrid comanipulation.

Furthermore, concerning the comanipulation control modes, we can point out that both the shared controllers (parllel hybrid and external hybrid) outperform the classical control law (Figure 8B). Finally, as for the tele-operation case, the parllel hybrid is more accurate. The average linear error is *e*_*t*_ = 0.67 mm (1.32 and 1.49 mm for the classical and external hybrid methods, respectively), while the average angular error is *e*_*r*_ = 0.45° (6.67° and 3.07° for the classical and external hybrid methods, respectively). This conclusion is confirmed by a post-hoc Tukey HSD analysis to evaluate the relevance of the obtained statistical data. As reported in Table 1, the comparison of the different control modes is statistically relevant confirming that the parallel hybrid control law outperforms in term of accuracy the other control laws, in both tele-operation (Figure 8B) and comanipulation (Figure 8B).

#### 4.3.2 Trajectory

**FIGURE 9:**
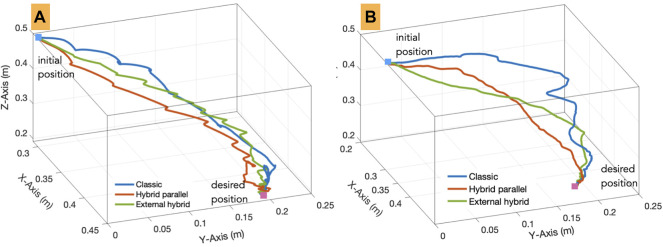
3D trajectories carried out by an operator using the implemented **(A)** tele-operation and **(B)** comanipulation modes.

The second evaluation criteria concerns the behavior of the robot end-effector while accomplishing the task (positioning and insertion). To do this, we have recorded the spatial trajectory performed by the 3D printed tool for each control mode. An example is shown in Figure 9A, which depicts the 3D trajectory performed by the tool tip for each of the three control tele-operation modes achieved by one subject. Figure 9B shows the trajectories achieved using the comanipulation modes. It can be pointed out that those executed by the parallel hybrid method are smoother compared to the others. This suggests that the parallel hybrid method is more intuitive and easier to handle. On the other hand, it appears that the major difference between the trajectories lies especially in the second phase of the task, i.e., insertion of the tool into the incision hole. Note that the insertion task is considered completed when the tool is inserted 5 mm into the simulated incision hole. In case of classical tele-operation and comanipulation methods, the operator has to repeat the process several times with small movements to find the correct orientation of the tool with respect to the incision hole before starting the insertion, while with the developed hybrid methods the operator can achieve this insertion with minimum effort.

#### 4.3.3 Behaviour

**FIGURE 10:**
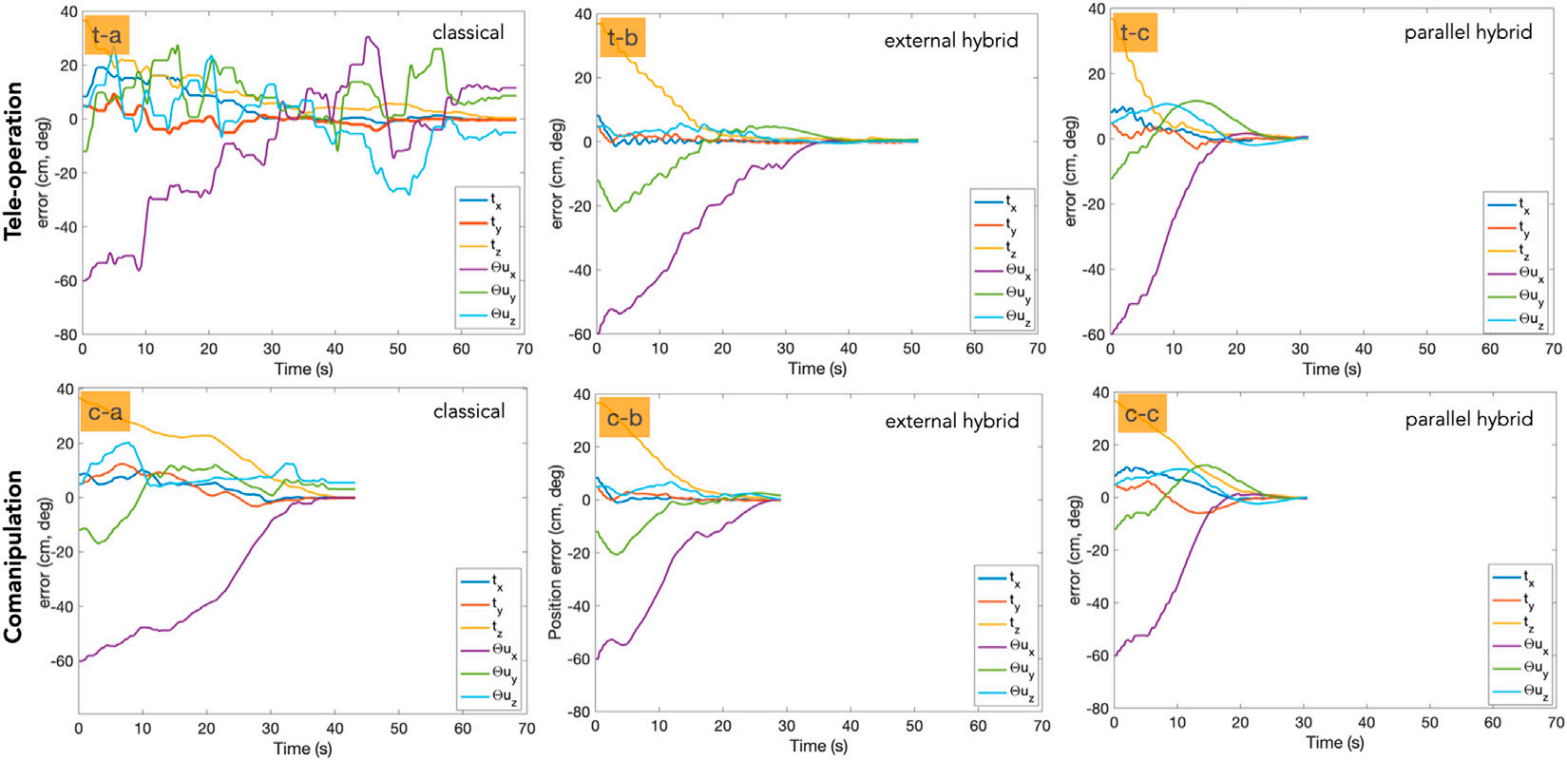
Error decay with each of the control modes. The first row shows the tele-operation mode and the second row represents the comanipulation mode.

The third evaluation criteria concerns the behaviour of the controllers according to the quality of the error **e** regulation as well as the velocities **v** sent to the robot while accomplishing the task. As can be seen in Figure 10 for teleoperation, the regulation to zero of the error in each DoF is smoother in the case of hybrid tele-operation methods (Figure 10 (t-b) (t-c)) compared to the classical one (Figure 10 (t-a)). In addition, both the hybrid controllers converged to zero, when the classical mode shows a significant residual error in several DoFs. The same observation can be made for the comanipulation evaluation as depicted in Figure 10 (right column). In contrast to the classical comanipulation (Figure 10 (c-a)), the hybrid comanipulation methods show smoother and close to exponential decay of the error (Figure 10 (c-b) (c-c)).

**FIGURE 11:**
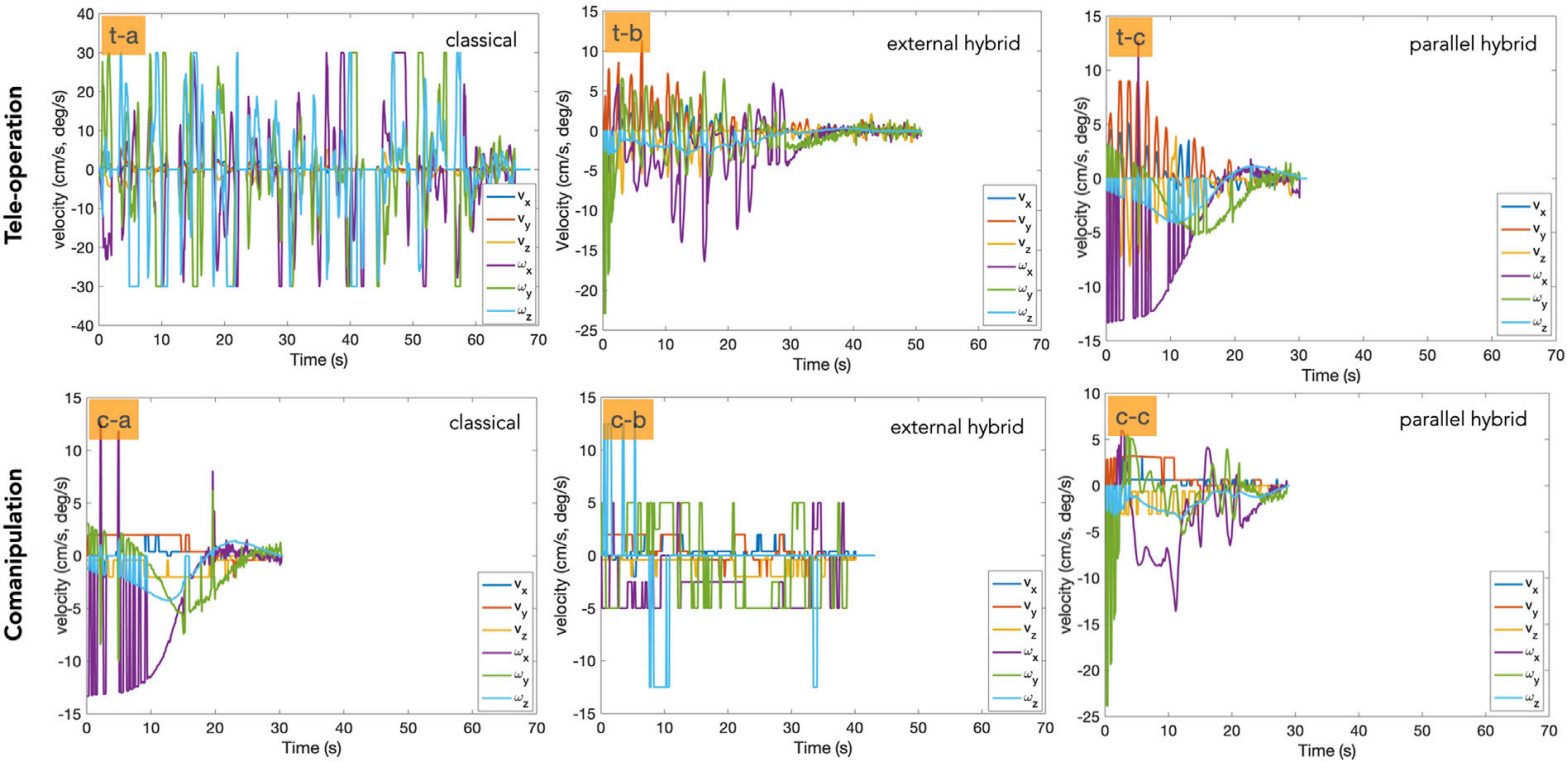
Illustration of the velocity twist involved in each control mode. The first row shows the tele-operation mode and the second row column represents the comanipulation one.

We have also analysed the velocity twist **v** sent to the robot. Figure 11 (left column) depicts the velocity twist evolution with tele-operation controllers, while Figure 11 (right column) shows the same for comanipulation modes. In the case of tele-operation, the hybrid approaches (Figure 11 (t-b) (t-c)) obviously show better behavior compared to the classical ones (Figure 11 (t-a)). Additionally, it can be noticed that the parllel hybrid methods (Figure 11 (c-c)) outperform both the classical and the external hybrid ones (Figure 11(c-a) (c-b)).

#### 4.3.4 Time Required to Achieve the Task

It was observed that the total time required to accomplish the task varies significantly from the hybrid methods comparing to the classical ones, for both the tele-operation and comanipulation modes. Table 2 summarizes the observed average time. It appears that the parllel hybrid tele-operation approach requires in average 40.53 ± 10.09 s, which means 50% faster than the classical and external hybrid ones. The same conclusion can be made for the comanipulation methods. Indeed, the parllel hybrid controller requires on average 29.03 ± 6.94 s to achieve the task, which means approximately 25*%* faster than the others control schemes.

**TABLE 2:** Time required to achieve the task with different control schemes.

Control method	Time duration (s)
Classical tele-operation	64.05 ± 33.84
Parallel hybrid tele-operation	***40.53*** ***±*** ***10.09***
External hybrid tele-operation	67.82 ± 26.53
Classical comanipulation	39.22 ± 8.38
Parallel hybrid comanipulation	***29.03*** ***±*** ***6.94***
External hybrid comanipulation	36.53 ± 5.40

Bold represents the best performance obtained in terms of accuracy on all degrees of freedom (translations and rotations) when comparing the different proposed control modes.

## 5 Conclusion

In this paper, we discussed new shared control schemes in both tele-operation and comanipulation modes. The objective was to provide the surgeon with an intuitive, efficient, and precise control approach for minimally invasive surgery using a robotic system. We presented six control schemes: a classical end-frame tele-operation, two shared vision/position tele-operation controllers (called external/parallel hybrid methods), a classical comanipulation controller, and two shared vision/force comanipulation controllers (also called external and parallel hybrid methods). In order to experimentally analyse the performances of the controllers and compare them to each other, we considered various evaluation criteria: accuracy, Cartesian trajectory of the tool tip, and time required to achieve the implemented positioning task. This evaluation showed that shared controllers (external hybrid and parallel hybrid) outperformed classical methods in both modes, i.e., tele-operation and comanipulation. In addition, the parallel hybrid approaches are much more efficient than the external hybrid ones in almost all the evaluation criteria.

Future work will focus on the implementation of the proposed controllers in real conditions of use, i.e., minimally invasive surgery in middle ear. Senior and junior surgeons will be recruited to evaluated the benefit of such approaches in the operating room.

## Data Availability

The raw data supporting the conclusion of this article will be made available by the authors, without undue reservation.
